# P7C3 Compounds as Targeted Mitochondrial Therapeutics for Brain Disorders

**DOI:** 10.1002/cns.71139

**Published:** 2026-09-05

**Authors:** Yajing Chen, Xiaohuan Du, Fang Li, Shuwei Yuan, Wenjing Wang, Zengyan Zhu, Mei Wang, Chao Gu

**Affiliations:** ^1^ Department of Pharmacy Children's Hospital of Soochow University Suzhou China

**Keywords:** drug discovery, mitochondrial quality control, molecular mechanisms, NAD^+^, NAMPT activator, neurological disorders, P7C3, targeted therapy

## Abstract

**Background:**

P7C3 compounds are aminopropyl carbazole derivatives identified through phenotypic screening for proneurogenic activity. They directly activate nicotinamide phosphoribosyltransferase (NAMPT), the rate‐limiting enzyme in the NAD^+^ salvage pathway. However, a comprehensive synthesis of their mechanisms and therapeutic potential across neurological disorders is currently lacking.

**Methods:**

A systematic literature review was conducted in PubMed, Web of Science, and Scopus to synthesize the discovery trajectory, structure–activity relationships, molecular mechanisms, and preclinical efficacy of P7C3 compounds. The following keyword combinations were used: (“P7C3” OR “P7C3 compound”) AND (“NAMPT” OR “NAD+” OR “sirtuin” OR “mitochondria”) AND (“neuroprotection” OR “neurodegenerative”).

**Results:**

P7C3 elevates intracellular NAD^+^ levels, engages SIRT1 and SIRT3 deacetylase cascades, enhances mitochondrial quality control and attenuates oxidative stress. This review discusses the discovery, structure–activity relationships, and molecular mechanisms of P7C3, with a particular emphasis on mitochondrial dynamics and redox homeostasis. The efficacy of P7C3 in preclinical studies was evaluated across Alzheimer's disease (AD), Parkinson's disease (PD), traumatic brain injury (TBI), ischemic stroke, depression, and chemotherapy‐induced neuropathy. These neuroprotective effects occur independently of disease‐specific aggregates. Challenges hindering clinical application include the on‐target safety of NAMPT activation given the concurrent development of NAMPT inhibitors for tumorigenesis, the absence of validated predictive biomarkers, and the failure of prior NAMPT‐targeting trials.

**Conclusions:**

P7C3 illustrates how phenotypic screening coupled with target deconvolution can yield therapeutic candidates with potential applications across a broad spectrum of neurological disorders.

## Graphical abstract

P7C3 activates NAMPT, the rate‐limiting enzyme in the NAD^+^ salvage pathway, elevating NAD^+^ levels in neurons. This engages SIRT1‐dependent nuclear signaling and SIRT3‐dependent mitochondrial regulation, enhancing mitochondrial quality control, reducing oxidative stress, and promoting neuronal survival. P7C3 demonstrates broad preclinical efficacy across Alzheimer's disease, Parkinson's disease, traumatic brain injury, and other neurological disorders.

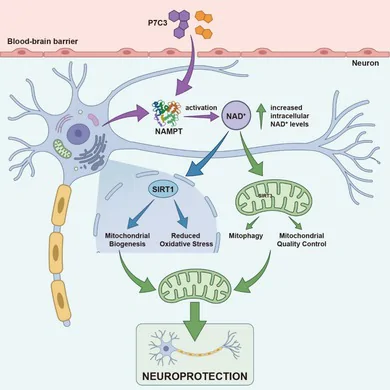

## 1 Introduction

Neurological disorders are a leading cause of disability worldwide, yet disease‐modifying therapies remain scarce. A central challenge in their treatment lies in the etiological heterogeneity of these conditions [1]. ad, PD, TBI, and ischemic stroke arise from distinct primary insults but converge upon shared downstream pathologies, most notably mitochondrial dysfunction, oxidative stress, and impaired proteostasis [2]. Mitochondria play central roles in neuronal energy metabolism and stress signaling [3]. Therefore, organelle‐directed strategies represent a rational therapeutic approach that can converge the treatment of individual diseases. These convergent pathways represent shared vulnerability nodes. Targeting these nodes may offer broader clinical utility than addressing disease‐specific triggers alone.

Mitochondrial integrity is robustly linked to cellular NAD^+^ availability. NAD^+^ serves as an obligate cofactor for sirtuin deacetylases, linking the metabolic state to adaptive transcriptional programs [4]. Mitochondrial SIRT3 modulates the function of key metabolic enzymes, including long‐chain acyl‐CoA dehydrogenase (LCAD) [5], isocitrate dehydrogenase 2 (IDH2) [6], and superoxide dismutase 2 (SOD2) [7], while nuclear SIRT1 coordinates biogenesis and antioxidant defenses via PGC‐1α [8] and FOXO3a [9]. The bioavailability of NAD^+^ is governed by nicotinamide phosphoribosyltransferase (NAMPT), the rate‐limiting enzyme of the salvage pathway [10]. NAD^+^ levels decline with aging and in neurodegenerative diseases, implicating NAMPT insufficiency in mitochondrial collapse [11]. Therefore, the pharmacological restoration of NAD^+^ represents a compelling strategy to reinforce neuronal resilience.

A phenotypic screening for proneurogenic compounds identified P7C3, an aminopropyl carbazole derivative [12]. This discovery illustrates the power of phenotypic screening coupled with target deconvolution, a paradigm that prioritizes functional outcomes over predetermined mechanisms. Subsequent studies indicated that P7C3 directly binds to and activates NAMPT, upregulating NAD^+^ levels and initiating sirtuin‐mediated protective cascades [13]. In this review, we synthesized the discovery trajectory, medicinal chemistry evolution, and molecular pharmacology of P7C3, with a particular focus on its coordinated regulatory effect on mitochondrial quality control and redox homeostasis. Preclinical evidence supporting the efficacy of P7C3 was critically appraised across a spectrum of neurological conditions, including AD, PD, TBI, ischemic stroke, depression, and chemotherapy‐induced neuropathy. Finally, we dissected the principal obstacles hindering the clinical translation of P7C3 and outlined priorities for future research.

## 2 Drug Discovery and Design

### 2.1 Phenotypic Discovery and Target Identification

In 2010, Pieper and colleagues conducted an unbiased phenotypic screening to identify small molecules capable of enhancing the survival of newborn neurons in the hippocampal dentate gyrus of adult mice [12]. This functional endpoint was chosen for its direct relevance to neuroprotection and required no prior assumptions regarding the underlying molecular mechanisms. The screening yielded 1‐anilino‐3‐(3,6‐dibromocarbazol‐9‐yl)propan‐2‐ol, also known as P7C3. It is an aminopropyl carbazole derivative with sufficient oral bioavailability, efficient blood‐brain barrier penetration, and a favorable safety profile at doses well above the minimally effective range [14]. Notably, the molecular target of P7C3 remained undefined for several years following its discovery. However, this mechanistic ambiguity did not prevent its extensive preclinical validation across diverse neurological diseases [15, 16, 17].

In 2014, Wang et al. identified nicotinamide phosphoribosyltransferase (NAMPT) as the direct molecular target of P7C3 [13]. They revealed that P7C3 directly binds to NAMPT and dose‐dependently enhances its enzymatic activity, with no change in the protein expression or stability of NAMPT [13]. This interaction results in a sustained increase in the intracellular levels of NAD^+^, an effect confirmed in both cultured primary neurons and the brains of treated mice. Elucidation of NAMPT as the molecular target provided a mechanistic foundation for subsequent optimization of the P7C3 scaffold.

### 2.2 Medicinal Chemistry Optimization

The evolution from the initial lead to optimized analogs with improved drug‐like properties exemplifies the iterative refinement characteristic of structure–activity relationship (SAR)‐guided medicinal chemistry. Systematic modification of the P7C3 scaffold delineated several structural determinants of its activity [14, 18, 19]. The carbazole core and the 3,6‐dibromo substitution pattern were shown to be essential for NAMPT binding and activation [13, 18]. Furthermore, it was found that the aminopropyl linker confers the conformational flexibility required for productive target engagement, while modifications to the terminal aniline moiety modulate potency, metabolic stability, and blood‐brain barrier penetration.

Among the optimized derivatives, (−)‐P7C3‐S243, the S‐enantiomer of the parent compound, offers improved metabolic stability, enhanced brain exposure, and a prolonged half‐life compared to the racemic mixture [19]. This analog has demonstrated robust protective effects in preclinical models of PD [20] and TBI [21]. P7C3‐A20 was synthesized through further structural refinement, resulting in enhanced neuroprotective potency and an improved toxicity profile [14]. Importantly, a scalable, chromatography‐free synthetic route was established for P7C3‐A20, enabling multi‐gram production to support extensive preclinical evaluation [22]. Collectively, P7C3 analogs exhibited favorable drug‐like attributes that distinguish them from conventional compounds. These features include oral bioavailability, efficient blood‐brain barrier penetration, and brain‐to‐plasma ratios compatible with central nervous system applications. Besides, P7C3 analogs exhibit minimal toxicity at doses substantially exceeding the therapeutic range, and their scalable synthesis facilitates both preclinical advancement and potential clinical development [23].

### 2.3 Additional Molecular Targets and Polypharmacology

**FIGURE 1:**
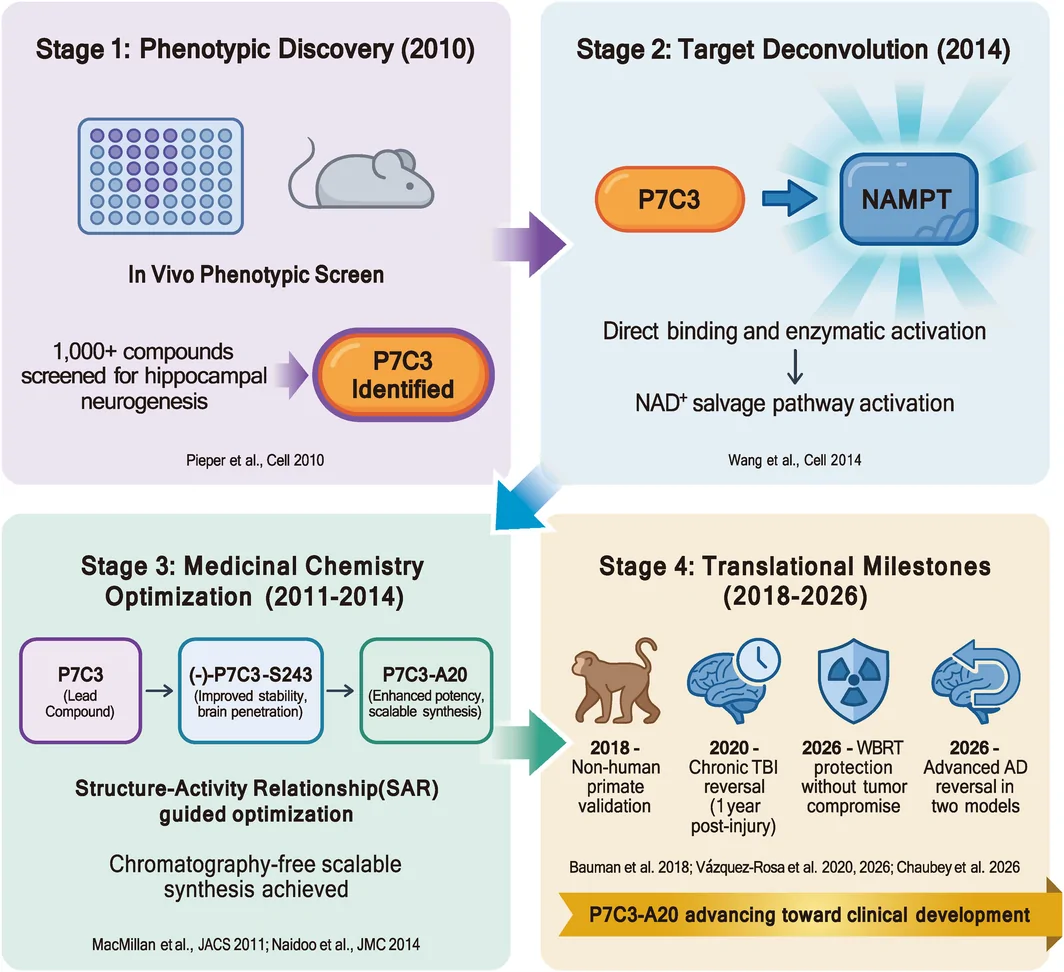
P7C3 drug discovery and development timeline. Schematic illustration depicting the four‐stage developmental process of P7C3: (1) Phenotypic screening identified P7C3 as a proneurogenic compound; (2) Target deconvolution found NAMPT as the direct molecular target of P7C3; (3) Medicinal chemistry optimization yielded (−)‐P7C3‐S243 and P7C3‐A20 with improved drug‐like properties and scalable synthesis; (4) Key translational milestones included nonhuman primate validation, reversal of chronic TBI one year after injury, protection against whole‐brain radiotherapy‐induced cognitive decline, and reversal of advanced AD pathology.

Although NAMPT is the primary and most extensively characterized target of P7C3, emerging evidence indicates that these compounds may engage additional proteins. Human protein microarray screening identified 577 candidate P7C3‐binding proteins, with global profiling indicating enrichment in glycolytic pathways. Subsequent validation in glioma cells confirmed that P7C3 directly targets phosphoglycerate kinase 1 (PGK1) at specific lysine and asparagine residues, thereby suppressing aerobic glycolysis and inhibiting malignant proliferation [24]. In hepatic stellate cells, P7C3 was shown to bind to eukaryotic translation initiation factor 4A1 (eIF4A1), thereby modulating protein translation and autophagic flux and reducing the expression of fibrosis markers, including collagen Type I alpha 1 (COL1A1) and fibronectin [25]. It should be noted that PGK1 and eIF4A1, while validated as P7C3‐binding proteins in cancer cells and hepatic stellate cells, have not yet been studied in neurons or glial cells. Whether these interactions occur in the nervous system or contribute to the neuroprotective effects of P7C3 remains unclear. Future studies using neuronal‐specific knockout or knockdown of these targets are warranted to dissect their contribution relative to NAMPT. Nevertheless, this emerging polypharmacology may contribute to the broad protective properties of P7C3 across diverse diseases. The drug discovery and development trajectory of P7C3 is illustrated in Figure 1.

## 3 Molecular Mechanisms

### 3.1 NAMPT Activation and Enhancement of NAD ^+^ Bioavailability

Nicotinamide phosphoribosyltransferase (NAMPT) catalyzes the conversion of nicotinamide to nicotinamide mononucleotide (NMN), the first and rate‐limiting step in the NAD^+^ salvage pathway. NMN is subsequently converted to NAD^+^ by nicotinamide mononucleotide adenylyltransferases (NMNATs), completing the biosynthesis of this essential cofactor [10]. P7C3 directly binds to NAMPT and enhances its enzymatic activity without affecting the protein expression or stability of NAMPT [13]. NAMPT activation is sufficient to drive a sustained increase in the intracellular concentration of NAD^+^. In primary cultures subjected to oxidative stress, treatment with P7C3 prevented the precipitous decline in NAD^+^ levels [13]. Consistent with the in vitro findings, systemic administration of P7C3 to aged mice subjected to TBI elevated NAD^+^ levels in the hippocampus, effectively normalizing them to levels observed in healthy young adults [17, 26]. Notably, P7C3 restored NAD^+^ homeostasis without inducing supraphysiological accumulation, a property that distinguishes it from other NAD^+^‐boosting strategies [27].

### 3.2 SIRT1‐Dependent Nuclear Signaling

SIRT1 is a nuclear NAD^+^‐dependent deacetylase activated by P7C3‐mediated increases in the intracellular levels of NAD^+^. In diabetic hearts, P7C3 upregulated the function of both NAMPT and SIRT1 [28]. In AD models, P7C3 promoted the NAD^+^‐SIRT1 pathway and enhanced mitophagy [29]. In a brain injury model, P7C3‐A20 downregulated acetylated tau, an effect linked to NAD^+^‐SIRT1‐dependent neuroprotection [30]. The transcription factor FOXO3a is a key substrate of SIRT1 engaged by P7C3. In a preclinical model of diabetic stroke, P7C3‐A20 restored the intracellular levels of NAMPT, SIRT1, and FOXO3a, attenuating the downregulatory effects induced by high‐glucose and oxygen–glucose deprivation [31]. The NAMPT‐SIRT1‐FOXO3a axis has been identified as a protective factor in diabetic stroke. In cerebral ischemia induced by high‐glucose conditions, P7C3‐A20 also suppressed high‐glucose and oxygen–glucose deprivation (HGC/OGD)‐induced increases in the protein levels of FOXO3a [32]. The tumor suppressor p53 represents another downstream effector modulated by P7C3. In PD models, P7C3 inhibited GSK3β activation, thereby suppressing p53 activity and attenuating Bax upregulation [33]. The GSK3β‐p53‐Bax pathway converges with NAD^+^‐SIRT1 signaling to further inhibit apoptosis. SIRT1‐mediated deacetylation of p53 may further contribute to this protective effect, although direct evidence in P7C3‐treated neurons is required.

### 3.3 SIRT3‐Dependent Mitochondrial Regulation

SIRT3 is the primary NAD^+^‐dependent deacetylase existing in the mitochondrial matrix. A P7C3‐mediated increase in NAD^+^ concentration activates SIRT3. Subsequently, SIRT3 deacetylates key mitochondrial enzymes, including superoxide dismutase 2 (SOD2), isocitrate dehydrogenase 2 (IDH2), and electron transport chain components, thereby enhancing ROS detoxification, NADPH production, and ATP synthesis [34]. In astrocytes, P7C3 upregulates the protein levels of SIRT3, enhances mitophagy, and downregulates mitochondrial ROS levels [35]. In microglia, P7C3‐A20 protects against OxyHb‐induced mitochondrial damage and suppresses neuroinflammation in a SIRT3‐dependent manner, with *SIRT3* conditional knockout abolishing these protective effects [36].

The engagement of SIRT3 by P7C3 has been validated in several disease models. In a whole‐brain radiotherapy model, P7C3‐A20 prevented chronic hippocampal oxidative stress and cognitive decline without impairing antitumor efficacy [37]. In an intracerebral hemorrhage model, P7C3‐A20 was shown to minimize lesion volume and maintain the integrity of the blood‐brain barrier despite damage through the NAD^+^‐SIRT3 pathway [36]. A recent study also indicated that the circadian gene *Period3* regulates the NAD^+^‐SIRT3 axis by binding to E‐box elements in the *Nampt* intronic region with BMAL1. Additionally, P7C3‐A20 administration recapitulated the protective effects of NAD^+^ supplementation in *Period3* knockout mice [38].

### 3.4 Mitochondrial Quality Control

P7C3 enhances mitochondrial quality control through coordinated regulation of biogenesis and mitophagy. The SIRT1‐PGC‐1α axis promotes mitochondrial biogenesis. In aged oocytes, treatment with P7C3 increased mitochondrial mass and respiratory capacity, with SIRT1 identified as a central regulator of the NAD^+^ regulatory network [26]. NAD^+^‐PINK1‐dependent mechanisms restore mitophagy, defined as the selective clearance of damaged mitochondria. P7C3‐mediated NAMPT activation was found to rescue mitophagic flux in diabetic keratopathy via this pathway [39]. In AD models, P7C3 enhanced hippocampal mitophagy and improved cognitive function [29]. P7C3 also preserved mitochondrial dynamics. In senile osteoporosis, P7C3 prevented the senescence of bone marrow mesenchymal stem cells by improving mitochondrial function through the NAMPT‐OPA1 axis, maintaining mitochondrial fusion and membrane potential [40].

### 3.5 Autophagy, Apoptosis, and Neuroinflammation

**FIGURE 2:**
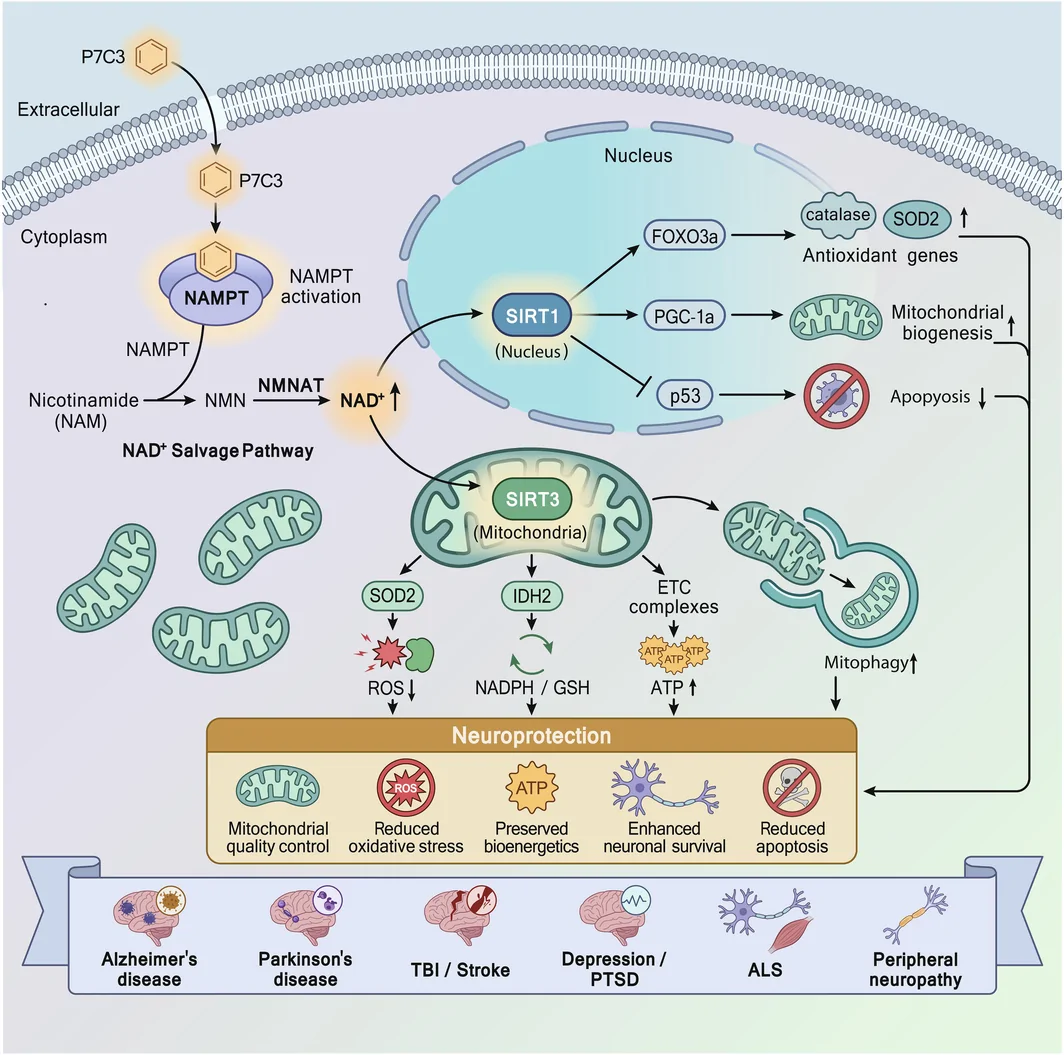
Molecular mechanisms of P7C3‐mediated neuroprotection. P7C3 enters neurons and directly binds to NAMPT, thereby enhancing the conversion of nicotinamide to NMN and upregulating intracellular NAD^+^ levels. Elevated NAD^+^ levels activate nuclear SIRT1 and mitochondrial SIRT3. SIRT1 deacetylates FOXO3a to promote the expression of antioxidant genes. SIRT1 also deacetylates PGC‐1α and p53 to enhance mitochondrial biogenesis and suppress apoptosis, respectively. SIRT3 deacetylates and activates SOD2 to prevent mitochondrial ROS accumulation. Furthermore, SIRT3 activates IDH2 to support NADPH production and glutathione regeneration. Moreover, SIRT3 enhances ETC complex activity to maintain ATP synthesis. P7C3 also restores PINK1‐dependent mitophagy and normalizes autophagic flux. Collectively, these effects preserve mitochondrial quality control, suppress oxidative stress, and promote neuronal survival in several neurological disorders.

P7C3 regulates autophagic flux in a context‐dependent manner. In TBI, P7C3‐A20 attenuated excessive autophagy and preserved basal autophagic activity [41]. In cerebral ischemia, P7C3‐A20 suppressed HGC/OGD‐induced increases in FOXO3a and LC3‐II levels, implicating the FOXO3a/LC3 axis in autophagy modulation [32]. In PD models, P7C3 protected dopaminergic neurons against MPP^+^‐induced death by inhibiting the GSK3β‐p53‐Bax pathway [33]. In congenital hypothyroidism, P7C3 suppressed the function of RIPK3, a key mediator of necroptosis [42]. P7C3 also attenuated neuroinflammation through multiple mechanisms. In microglia, P7C3 suppressed LPS‐induced expression of IL‐1β and TNF‐α by inhibiting the NF‐κB pathway, thereby preventing dopaminergic neuronal loss [43]. In astrocytes, P7C3 suppressed senescence and inhibited the release of senescence‐associated secretory phenotype (SASP) factors, including IL‐6, IL‐1β, CXCL10, and MMP9, through Nrf2/SIRT3‐mediated activation of mitophagy [35]. The molecular mechanisms underlying P7C3‐mediated neuroprotection are summarized in Figure 2.

## 4 The Preclinical Efficacy of P7C3 Across Brain Disorders

### 4.1 AD

Chronic treatment with P7C3‐S243 improved cognitive function and depressive‐like behaviors in the TgF344‐AD rat model [44]. These functional benefits occured without detectable reductions in amyloid‐β plaques or tauopathy [44]. This dissociation indicates that P7C3 preserves neuronal function independently of the removal of canonical AD aggregates. This resilience‐based mechanism may offer therapeutic benefits even in patients with established pathology and could be applicable across multiple neurodegenerative conditions that share mitochondrial dysfunction as a common downstream vulnerability. In APP/PS1 mice, P7C3 promoted the NAD^+^‐SIRT1/3 axis and enhanced hippocampal mitophagy, and these changes were correlated with improved learning and memory functions [29].

A landmark study also revealed that P7C3‐A20 reverses advanced pathological changes induced by AD in aged 5xFAD mice [45]. P7C3‐A20 reduced plaque deposition, tau hyperphosphorylation, blood‐brain barrier disruption, and synaptic deficits and restored behavioral performance [45]. In proteomic analyses, P7C3‐A20 corrected aberrant protein signatures in 5xFAD mice, many of which overlapped with alterations in postmortem human AD brain [45]. Notably, the efficacy of P7C3‐A20 was observed in both an Aβ‐driven model and a tauopathy model [45]. This dual validation suggests that P7C3‐induced NAD^+^ restoration targets convergent downstream pathways rather than disease‐specific triggers.

### 4.2 PD

Dopaminergic neurodegeneration in PD is driven by mitochondrial dysfunction, oxidative stress, and neuroinflammation [46]. P7C3‐S243 protected dopaminergic neurons, preserved striatal dopamine content, and improved motor function in the 6‐OHDA mouse model [20]. Similarly, aminopropyl carbazoles blocked MPTP‐induced dopaminergic neuron loss in the substantia nigra [15]. Mechanistically, these protective effects are mediated through the stabilization of mitochondrial membrane potential and GSK3β inhibition, which in turn suppresses p53 activity and attenuates Bax‐mediated apoptosis [33].

P7C3 also protects dopaminergic neurons by modulating glial responses. In the MPTP model, P7C3 suppressed astrocyte senescence by enhancing Nrf2/SIRT3‐mediated mitophagy, thereby preventing the development of the senescence‐associated secretory phenotype [35]. P7C3 also inhibited microglial activation via the NF‐κB pathway and subsequently attenuated the release of IL‐1β and TNF‐α [43]. These combined actions on neurons and glial cells highlight the therapeutic potential of P7C3 in PD.

### 4.3 TBI

TBI is a leading cause of death and long‐term disability worldwide. P7C3 compounds have shown protective effects in both acute and chronic TBI models. In acute experimental TBI, P7C3‐A20 preserved cortical neurons and improved sensorimotor and cognitive function [17]. In blast‐mediated TBI, P7C3‐S243 initiated one day after injury, blocked axonal degeneration and preserved synaptic activity, motor coordination, and learning and memory functions [47]. These acute benefits were mediated partly through suppression of excessive autophagy and apoptosis [41].

Notably, the therapeutic effects of P7C3 extend beyond the acute phase. In a pivotal 2020 study, treatment with P7C3‐A20 initiated one year after TBI repaired the integrity of the blood‐brain barrier, inhibited ongoing axonal degeneration, and restored the cognitive performance of mice [48]. These enduring benefits were accompanied by increased pericyte coverage, elevated expression of tight junction proteins, and suppressed neuroinflammation [48, 49]. The efficacy of P7C3 in chronic conditions suggests that NAD^+^ restoration can reactivate regenerative processes even long after injury.

### 4.4 Ischemic Stroke

Ischemic stroke is a leading cause of death and disability worldwide, with limited effective therapeutic strategies. P7C3‐A20 has shown protective effects in several stroke models. Following transient middle cerebral artery occlusion in rats, P7C3‐A20 improved sensorimotor function and cognitive performance while preventing cortical and hippocampal atrophy [50]. These benefits were accompanied by increased hippocampal neurogenesis and restoration of cortical NAD^+^ levels [50]. Notably, delayed treatment initiated six hours after reperfusion still effectively reduced infarct volume and preserved functional outcomes [51]. P7C3‐A20 also protected against diabetes‐associated stroke. In mice with Type 2 diabetes subjected to cerebral ischemia, P7C3‐A20 preserved cell viability and restored the NAMPT/SIRT1/FOXO3a pathway [31]. These findings highlight the role of the NAMPT/SIRT1/FOXO3a pathway as a critical protective axis disrupted by diabetes‐associated stroke. In a humanized cerebral organoid model of OGD, P7C3‐A20 also exhibited neuroprotective effects [52].

### 4.5 Amyotrophic Lateral Sclerosis (ALS) and Spinal Cord Injury (SCI)

ALS and SCI are devastating neurological diseases characterized by progressive motor neuron loss and permanent functional deficits, respectively. Effective therapeutic regimens that prevent neurodegeneration or promote neural repair remain limited.

In the SOD1‐G93A mouse model of ALS, treatment with P7C3 blocked spinal motor neuron death, significantly extended survival, and delayed motor decline [16]. In the rat model of SCI, P7C3 promoted functional recovery, decreased lesion volume, and enhanced neuronal survival and myelination [53]. The combination of P7C3‐A20 and collagen hydrogel further promoted neuronal differentiation of endogenous neural stem cells and enhanced functional recovery [54]. Additionally, P7C3‐A20 was incorporated into small‐molecule cocktails capable of reprogramming fibroblasts into functional induced neurons, highlighting its potential in cell replacement strategies [55].

### 4.6 Depression and Post‐Traumatic Stress Disorder

Depression and post‐traumatic stress disorder (PTSD) are common neuropsychiatric disorders characterized by hippocampal dysfunction, impaired neurogenesis, and synaptic loss. In both conditions, P7C3 ameliorated behavioral deficits in preclinical models. In the chronic social defeat stress model, treatment with P7C3 reversed stress‐induced social avoidance and anhedonia [56]. This effect was abolished after hippocampal irradiation, confirming the role of P7C3‐induced neurogenesis [56]. P7C3 restored impaired fear extinction in a genetic PTSD model with *CACNA1C* deficiency in dopamine D1 receptor‐expressing neurons [57]. P7C3‐A20 administration during the gestational period mitigated anxiety‐like and depression‐like phenotypes in adult offspring and preserved maternal behavior under chronic stress [58, 59].

### 4.7 Peripheral Neuropathy, Retinal Protection, and Other Neurological Applications

P7C3 was shown to protect the peripheral nervous system and sensory organs. It attenuated paclitaxel‐induced peripheral neuropathy without compromising the efficacy of chemotherapeutic agents [60]. Treatment with P7C3 preserved retinal ganglion cell viability following optic nerve crush injury [61]. P7C3 facilitated functional recovery after neonatal peripheral nerve injury [62]. Validation in nonhuman primates reinforced the therapeutic relevance of these findings, with P7C3 augmented hippocampal neurogenesis and promoted the expression of synaptic proteins in rhesus macaques [63].

Additional benefits have been observed in other neurological disorders. In neonatal hypoxic‐ischemic encephalopathy, P7C3‐A20 reduced infarct volume by activating the PI3K/AKT/GSK3β pathway [64]. In addition, P7C3 attenuated neuronal DNA damage associated with congenital hypothyroidism [65]. In infection‐related delirium, P7C3‐A20 prevented lipopolysaccharide‐induced electroencephalographic abnormalities [66]. Besides, P7C3 suppressed Th17 cell differentiation in experimental autoimmune encephalomyelitis [67]. Inflammatory pain models also unfolded the GABAergic antinociceptive activity of P7C3 [68].

### 4.8 Beyond the Nervous System

P7C3 exhibited protective effects in multiple organs and systems beyond the central nervous system. In bone, it counteracted bone loss by inhibiting osteoclast differentiation and promoting osteogenesis [69]. Furthermore, it attenuated the damage induced by postmenopausal osteoporosis or ionizing radiation [69, 70]. In skeletal muscle, P7C3 improved metabolic function in diabetic models [71] and accelerated recovery following injury [72]. P7C3 analogs also revealed cardioprotective effects in diabetic hearts subjected to ischemia‐reperfusion injury [28]. In the liver, P7C3‐A20 alleviated fatty liver disease by modulating the composition of the gut microbiota [73]. P7C3 also ameliorated hepatic fibrosis by targeting eIF4A1‐mediated protein translation and autophagy in hepatic stellate cells [25]. P7C3‐mediated NAMPT activation also attenuated acetaminophen‐induced acute liver injury [74].

## 5 Challenges and Future Perspectives

### 5.1 The Activation–Inhibition Dichotomy of NAMPT

A fundamental therapeutic dichotomy underlies NAMPT‐targeted interventions. NAMPT activation is pursued for neuroprotection, whereas NAMPT inhibition is concurrently regarded as an anticancer strategy since many tumors exhibit elevated NAMPT expression and depend on NAD^+^ availability for sustained proliferation [75]. This divergence necessitates caution regarding the long‐term safety of chronic NAMPT activation. In particular, whether sustained activation of NAMPT may increase the risk of cancer or prove contraindicated in patients with a history of malignancy remains to be investigated. Notably, higher concentrations of P7C3 were shown to suppress the growth and metastasis of certain types of cancer [76], indicating context‐dependent effects that warrant further systematic investigation. Long‐term toxicology studies specifically designed to assess oncogenic potential remain essential before long‐term administration in humans.

### 5.2 Safety Insights From Studies Focusing on NAMPT Inhibitors

Clinical experiences with NAMPT inhibitors offer instructive lessons for NAMPT‐targeted therapeutics [77]. Of the five NAMPT inhibitors evaluated clinically, three were discontinued due to safety concerns [77]. Common toxicities included dose‐limiting thrombocytopenia, gastrointestinal side effects, and retinal toxicity [78]. Retinal degeneration occurred rapidly in rodent models, manifesting as photoreceptor and outer nuclear layer loss [79]. These toxicities likely reflect the key role of NAD^+^ in highly proliferative and metabolically demanding tissues. Although safety considerations differ fundamentally between activators and inhibitors, sustained pathway activation may still perturb NAD^+^ homeostasis, warranting careful evaluation.

### 5.3 Predictive Biomarkers for Therapeutic Response

The heterogeneity of neurological disorders necessitates the stratification of patients most likely to benefit from NAMPT activation. Biomarkers include baseline NAD^+^ levels, NAMPT expression or enzymatic activity, and genetic variants of NAMPT or sirtuin pathway genes [4]. Notably, dopaminergic neurons in the substantia nigra express high levels of NAMPT. NAMPT deficiency alone triggered dopaminergic neuron loss and PD‐like motor dysfunction [80]. Circadian dysfunction also emerged as a particularly promising predictive marker. The circadian gene Period3 regulates the NAD^+^‐SIRT3 axis by enhancing NAMPT activity through E‐box binding to BMAL1 [38]. This suggests that patients with pre‐existing circadian impairment may have NAMPT dysregulation; therefore, they may benefit more from NAMPT activation.

### 5.4 Future Perspectives

Several advances may accelerate the development of P7C3‐based therapies. Exosomal NAMPT represents a dual‐purpose tool, functioning as both a delivery vehicle and a non‐invasive biomarker for monitoring target engagement [4]. The mechanistic coupling between circadian clock components and NAD^+^ metabolism suggests that chronotherapeutic dosing can synchronize P7C3 administration with endogenous NAD^+^ fluctuations to maximize therapeutic benefits [38]. Validation in humanized cerebral organoids [52] and nonhuman primates [63] strengthened confidence in the efficacy of P7C3 and reduced reliance on rodent models with limited predictive capacity. Nanoparticle‐based formulations offer an additional avenue to enhance drug delivery and achieve sustained release [81]. From a clinical translation perspective, chronic TBI and early‐stage AD represent logical entry points for first‐in‐human trials, with baseline NAD^+^ levels and circadian status as potential stratification biomarkers. Combination with nicotinic acid or NAD^+^ precursors may further optimize safety and efficacy.

## 6 Conclusion

P7C3 compounds highlight the value of target‐agnostic phenotypic screening followed by rigorous target deconvolution. As direct NAMPT activators, they upregulate NAD^+^ levels and engage the sirtuin signaling axis to reinforce mitochondrial quality control, suppress oxidative stress, and restore autophagic and mitophagic flux. These effects converge on fundamental vulnerability pathways shared across etiologically diverse neurological disorders. In recent studies, NAMPT activation reversed advanced Alzheimer's disease pathology in two distinct models, protected against radiotherapy‐induced cognitive decline without impairing cancer treatment, and exhibited robust efficacy against diabetic conditions. Validation in nonhuman primates also supported clinical translation; however, significant challenges still persist, including the on‐target safety implications of chronic NAMPT activation, the need for predictive biomarkers, and the absence of high‐resolution structural data to optimize next‐generation compounds. Addressing these obstacles may make P7C3 and its successors a new class of therapeutics that target the metabolic resilience of the brain in a broad spectrum of neurological disorders.

## Author Contributions

Yajing Chen: conceptualization, literature search, writing – original draft, figure preparation. Xiaohuan Du: literature search, data curation, writing – original draft. Fang Li: literature search, data curation. Shuwei Yuan: literature search, validation. Wenjing Wang: literature search, figure preparation. Zengyan Zhu: conceptualization, supervision, writing – review. Mei Wang: conceptualization, supervision, writing – review and editing. Chao Gu: conceptualization, supervision, writing – review and editing, project administration, funding acquisition. Zengyan Zhu, Mei Wang, and Chao Gu contributed equally as co‐corresponding authors. All authors reviewed and approved the final version of the manuscript.

## Funding

National Natural Science Foundation of China (Grant Number: 32200775); Jiangsu Innovative and Enterpreneurial Talent Programme (Grant Number: (2021) 31576); Suzhou Medical Health Science and Technology Innovation Project (Grant Number: SYW2025109); Suzhou Health Talent Plan Talent Research Project (Grant Number: GSWS2023102).

## Conflicts of Interest

The authors declare no conflicts of interest.

## Data Availability

Data sharing not applicable to this article as no datasets were generated or analyzed during the current study.
